# Directional analysis of intensity changes for determining the existence of cyst in optical coherence tomography images

**DOI:** 10.1038/s41598-022-06099-6

**Published:** 2022-02-08

**Authors:** Maryam Monemian, Hossein Rabbani

**Affiliations:** Medical Image & Signal Processing Research Center, School of Advanced Technologies in Medicine, Isfahan University of Medical Sciences, Isfahan, 8174673461 Iran

**Keywords:** Biomedical engineering, Computer science, Optics and photonics, Retinal diseases

## Abstract

Diabetic retinopathy (DR) is an important cause of blindness in people with the long history of diabetes. DR is caused due to the damage to blood vessels in the retina. One of the most important manifestations of DR is the formation of fluid-filled regions between retinal layers. The evaluation of stage and transcribed drugs can be possible through the analysis of retinal Optical Coherence Tomography (OCT) images. Therefore, the detection of cysts in OCT images and the is of considerable importance. In this paper, a fast method is proposed to determine the status of OCT images as cystic or non-cystic. The method consists of three phases which are pre-processing, boundary pixel determination and post-processing. After applying a noise reduction method in the pre-processing step, the method finds the pixels which are the boundary pixels of cysts. This process is performed by finding the significant intensity changes in the vertical direction and considering rectangular patches around the candidate pixels. The patches are verified whether or not they contain enough pixels making considerable diagonal intensity changes. Then, a shadow omission method is proposed in the post-processing phase to extract the shadow regions which can be mistakenly considered as cystic areas. Then, the pixels extracted in the previous phase that are near the shadow regions are removed to prevent the production of false positive cases. The performance of the proposed method is evaluated in terms of sensitivity and specificity on real datasets. The experimental results show that the proposed method produces outstanding results from both accuracy and speed points of view.

## Introduction

Optical Coherence Tomography (OCT) is an imaging modality which makes possible the process of capturing images from light-scattering organs such as retina. OCT technology uses light waves to take images of the retina. The high speed, high resolution and non-invasiveness of OCT have made a wide range of applications in the field of detecting and monitoring diseases^1,2^. OCT produces a large amount of data the manual processing of which is time-consuming and error-prone^3,4^.

**Figure 1:**
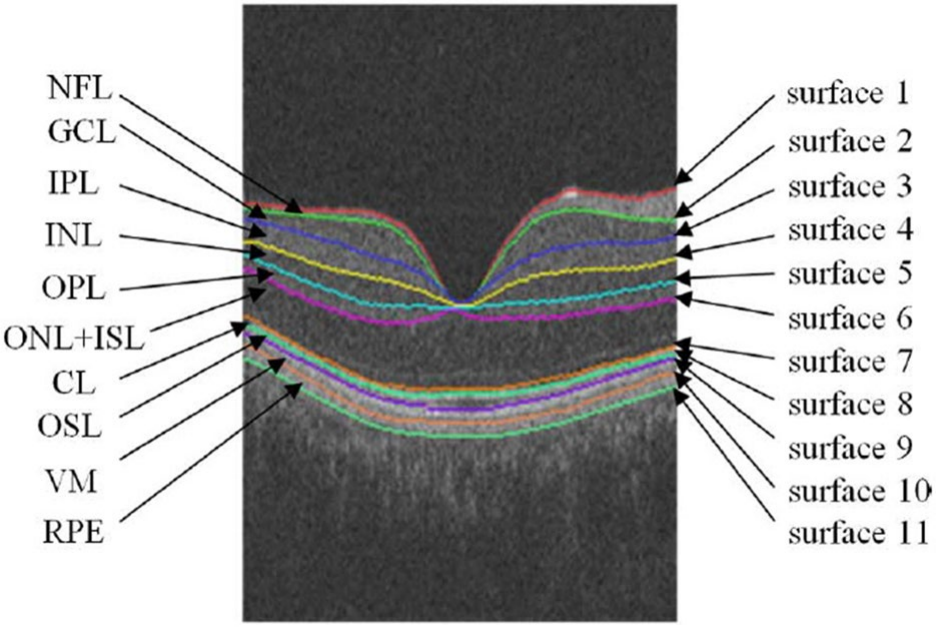
An OCT image including retinal layers and borders^5^.

Retina is an important organ of body the important diseases of which can threaten the human vision and even lead to blindness, if they left untreated. One beneficial tool for evaluating retinal status is OCT. Figure 1 presents an OCT image of retina^5^. Retina is a layered structure that each of layers has its own characteristics. The border numbers and layer names are presented in Fig. 1.

Some of the main retinal diseases are Diabetic Macular Edema (DME) and Age-related Macular Degeneration (AMD) the manifestations of which include fluid regions between retinal layers. The fluid-filled regions between retinal layers can be of different types, shapes and sizes. This makes the process of fluid regions detection and segmentation challenging. Thus, the identification of fluid-filled regions in the OCT images with short processing time and good accuracy is of considerable importance in the detection of retinal diseases and also the evaluation of effectiveness of drugs^6,7^.

For the purpose of cyst detection and segmentation in OCT retinal images, a number of works have been proposed^6–26^. The existing works can be categorized based on the approaches they used including graph theory^12,23,24^, artificial intelligence^8,9,15–17,25,26^, level set^14^ and knowledge^11,19^. Several sample works from each category are explained in the next section.

In addition to cyst detection and segmentation, in some applications it is only sufficient to determine whether or not the retinal images include cyst. In fact, the applications determine the necessity of detecting images with cyst or segmenting the existing cysts. In some cases, it may be necessary to find the boundaries of cyst for surgery purposes, determining the severity of disease and evaluating the size of cysts^27^. For these purposes, it is good to have the segmentation results. However, there are other applications where the ophthalmologists only want to find the first diagnosis of disease or utilize a semi-automatic method. In such cases, having the accurate boundaries of cysts does not help the ophthalmologists. Moreover, to obtain the exact boundaries may last a considerable period of time. Therefore, spending a non-neglectable period of time for providing unnecessary results is not reasonable.

In this paper, a new cyst detection method for retinal OCT images is proposed. This method firstly determines the boundary pixels for all types of fluid regions. This process can be performed by initially extracting distinguishing features for those boundary pixels. Then, a novel method is proposed for determining shadow columns in the OCT images. Since shadow regions are darker than background, they may produce boundary points which may be interpreted as boundary points for cysts. Therefore, this novel method helps in the omission of superfluous points falsely extracted as boundary pixels in the previous phase. One of the main benefits of the proposed method is to help ophthalmologists in the fast detection of cystic images and removing non-cystic images without the need to exact segmentation. The main contributions of this paper are summarized as follows.

- To determine whether or not an OCT B-scan includes cysts and to localize the approximate location of cysts with a high speed.
- To suggest a novel method for identifying shadow columns in OCT B-scans. This is helpful in reducing false positive points while preventing from the reduction of the true positive points.
- To detect cystic images without need to exact retinal layer and fluid segmentation.

The remainder of paper is organized as follows. The related works are explained in “Related works” section. “Method” section introduces the required preliminaries and the proposed method. The experimental results for the purpose of evaluation of the proposed method are presented in “Experimental results” section. Finally, “Conclusions” section includes the concluding remarks.

## Related works

In this section, the existing works related to the field of cyst detection and segmentation in OCT retinal images are explained.

A number of existing works used artificial intelligence-based approaches for the segmentation of cysts in OCT images^8,9,15–17,25,26^. In^8^ a Fully Convolutional Network (FCN) is used is suggested to segment intra-retinal cysts. The method includes a pre-processing step for removing noise, layer segmentation performed by Iowa reference method^28^ and cyst segmentation. After layer segmentation, Region of Interest (ROI) is determined and contrast enhancement operations are performed. Then, the segmented layers are considered as input for FCN and a prediction matrix is produced to reconstruct a binary mask corresponding to the cystoid regions of image^8^. In^9^ a cyst segmentation method is proposed which works based on the selective enhancement of cysts performed by a Convolutional Neural Network (CNN). The selective enhancement of cysts is performed by constructing Generalized Motion Patterns (GMP). Then, a function is made to enhance only cystoid regions using CNN. The output of CNN is a probability map in which the pixels which belong to cysts have higher probabilities than others^9^. Another deep learning approach called Relay-Net is suggested in^16^ for cyst segmentation in OCT images. The proposed network uses an encoder-decoder configuration where encoders including convolutional blocks learn contextual features and decoder convolutional blocks are used for semantic segmentation. Then, the network is trained to optimize a loss function^16^. In^17^ a FCN is developed for the segmentation and quantification of intra-retinal cysts in OCT B-scans from multi vendors. In the developed networks all pixels are separately analyzed and a probability of belonging to an intra-retinal cyst is assigned to each of them. A wide range of cysts including micro-cysts to large intra-retinal cysts can be segmented using the FCN of^17^. In^26^ a graph-cut algorithm is utilized to segment retinal layers and a FCN is trained to detect and label fluid pixels. Then, a random forest classifier is employed to remove the falsely labeled cystoid regions. In^15^ a method for simultaneous segmentation of retinal layers and fluids is proposed. At first, image-based features are extracted and a voxel classification is trained using the extracted features together with manual labels. The output of such a classifier is a probability map which is used both for surface segmentation and context-based feature extraction. Then, all the extracted features are used in training another classifier.

With respect to knowledge-based methods for cyst detection and segmentation, in^11^ the characteristics of cysts and retinal layer tissues are analyzed. A large set of features are defined for discriminating cysts from normal tissues. Then, a subset of the defined features is chosen using a feature selection algorithm. After determining the Region of Interest between ILM to RPE layers, square windows are considered and analyzed to verify the presence of cysts. For each window, a set of features such as Histogram of Oriented Gradients (HOG), Gabor filter, Local Binary Patterns are computed. Then, a feature selection strategy is utilized to choose the features that provide the highest distance between classes and a small variance within the classes. In^19^ a method for the identification of fluid-filled regions in OCT B-scans is proposed. At first, a Region of Interest which is between ILM and RPE layers is determined to limit the search space for cysts. The main idea utilized in^19^ is that the texture features of the fluid-filled regions differ from healthy regions. Therefore, an analysis should be performed on the different regions of the OCT image. The features of fluid-filled regions include intensity-based and texture-based feature. It is shown that the characteristics of fluid-filled regions show more homogeneous patterns compared to the characteristics of healthy tissues. Therefore, the analysis of homogeneity of characteristics can also help in finding the abnormal tissues. Then, a feature selection method is utilized to choose the ones with the highest discriminative power. The extracted features are used for training and testing in a classifier.

In^14^ a fuzzy level set method is proposed for the volumetric segmentation of cysts. At first, fuzzy C-means method is utilized to detect the fluid regions based on the fact that fluid regions are darker than retinal tissues. Then, level set method is applied on OCT B-scans and C-scans to detect fluid regions.

In^12,23,24^ several graph-based methods are suggested for the same purpose. In^12^ a graph-based approach is proposed for the segmentation of fluid-related abnormalities in OCT images. Firstly, the voxels that likely belong to the abnormalities are found according to the probabilities produced by a classifier. Then, retinal layers are segmented using graph-search method. In the second stage, graph-cut method which is good in the object segmentation is utilized for Symptomatic Exudate-Associated Derangement (SEAD) segmentation. In^24^ cyst segmentation is performed using a neutrosophic transform and graph-based shortest path. Firstly, the image is transformed into three sets including true, intermediate which represents noise and false. Then, graph-based shortest path method is applied to image in the neutrosophic space to segment Nerve Fiber Layer (NFL) and Retinal Pigment Epithelium (RPE) layer boundaries (presented in Fig. 1) to limit the Region of Interest (ROI). Then, a cost function for cyst segmentation is designed.

In contrast to the related existing works, our main purpose is to design a new cyst detection method which can identify the features of a cystoid OCT image with high speed to help ophthalmologists in removing non-cystoid images. In this regard, it is necessary to find the discriminating features of boundary pixels of cysts and look for pixels with such characteristics. Therefore, it is not necessary to consume time to segment retinal layers and fluids which inflicts a large volume of computations, too.

## Method

In this section, the proposed method is explained in details. All phases of method were performed in accordance with the relevant guidelines and regulations. The proposed method which is called Cyst Identification Based on Intensity Changes’ Analysis (CIBICA) consists of three main phases. In the first phase, the retinal OCT image is de-noised with the help of some de-noising methods. In the second phase, the points which are the boundary pixels of cysts with high probability are extracted based on the analysis of intensity changes in the OCT images. In the third phase, a new method is suggested to detect shadow columns in OCT images and remove the superfluous points extracted in the second phase. In the following, the application of the proposed method is introduced. Then, all the phases are explained in details.

### Application

The main application of our proposed method is for the diagnosis purposes. For instance, assume that there is no B-scan including cyst according to the result of proposed method. In this case, the proposed method helps the ophthalmologist to reject the hypothesis of diseases with cyst manifestations such as Cystic Macular Edema (CME).

In addition, in many cases, the ophthalmologists do not have sufficient time for verifying a large number of OCT B-scans and it is very helpful to present only the informative ones for them. This task is usually performed by operators who visually verify the images and choose some more important ones for the ophthalmologist. It should be noted that the operator may not have enough time, skill or knowledge and miss some informative images. Thus, the automatic selection of more informative images with high accuracy can be very advantageous.

In the literature, there are many research works which consider cysts as the important signs of salient retinal diseases^29^. In fact, cysts are important abnormalities the detection of which can help to control the progressive trend of dangerous disease. For instance, in^29^ the authors introduce subretinal pseudocysts as a manifestation of DME disease. Also, the fluid-filled regions are considered as the specific biomarkers for DME^30^. In^31^, it has been shown that elongated cystic regions can be observed in a large percent of AMD cases. The accumulation of fluid inside and underneath the retina may affect the structure of retinal layers and even lead to the loss of vision^32^. Since in many important retinal diseases, the abnormalities appearing in the B-scans include cysts^30,31^, it is very beneficial to search and find the images containing cysts.

In addition, it is necessary for the ophthalmologists to evaluate the effectiveness of drugs through monitoring cystic B-scans^33^. In other words, by extracting cystic images and removing non-cystic ones, they can save time in the evaluation process. Also, finding the cystic images and verifying them helps in the determination of best treatments for the ophthalmologists.

### Pre-processing

In the process of capturing OCT images, speckle noise is usually generated which in turn degrades the quality of images^34^. Since the proposed method works based on the analysis of intensity levels of the OCT image, it is necessary to decrease the noise before other steps. In this stage, the retinal OCT image is de-noised with the help of some noise reduction method. The noise reduction method which is employed here is Sparsity Based Simultaneous De-noising and Interpolation (SBSDI)^35^. The basis of SBSDI method for de-noising is the construction of a sparse representation dictionary which includes the relationship between several reference image pairs. Each image pair contains a low-quality image and a high quality image. SBSDI method can predict the missing information in the low-quality images with the help of the constructed dictionary^35^. Although the de-noising is performed using SBSDI method here, the performance of cyst detection of CIBICA method does not depend on the type of de-noising method.

### Boundary pixel determination

**Figure 2:**
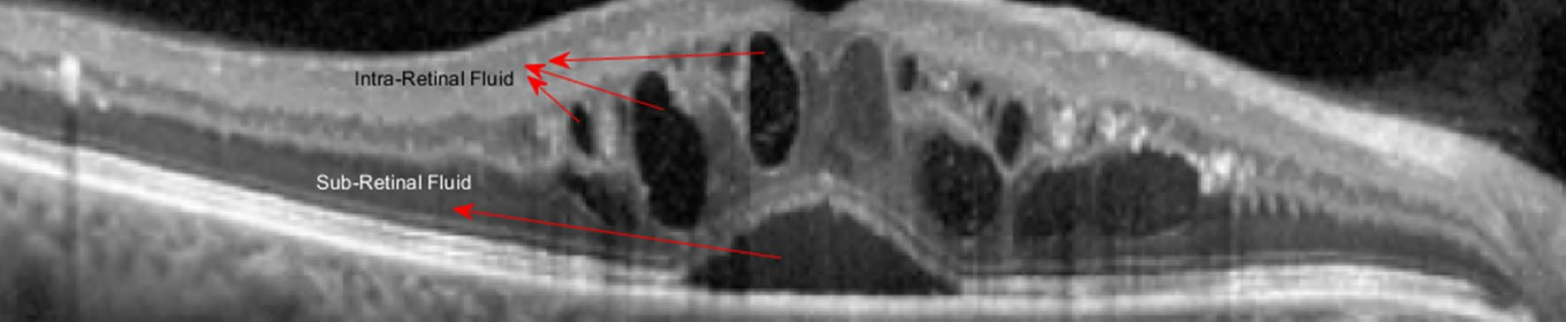
An OCT B-scan including SRF and IRF.

In this section, a new method is proposed for extracting the boundary pixels of cysts in the OCT images. At first, it should be noticed that a fluid-filled region in the retinal OCT image can be observed as a dark region formed between retinal layers or near the layer boundaries. The fluid-filled regions in the retina can be divided to Sub-Retinal Fluids (SRF) and Intra-Retinal Fluids (IRF). Both of them are presented in Fig. 2.

The basis for the boundary pixel determination phase is to find the vertical intensity changes during the image columns. Then, the points at which a significant vertical change is observed are determined. For each of these points, a rectangular patch is considered in the left and right sides. In the rectangular patch, we look for the points at which a significant diagonal change can be observed. If the patch includes a sufficient number of points with such a characteristic, the first point can be considered as a candidate for being a boundary pixel for cyst.

Let *I*_m*n_ denote an OCT image with *m* rows and *n* columns. Let ${p}_{i,j}$ and ${X}_{i,j}$ denote the pixel located at (*i*,*j*) in the OCT image and its intensity value, respectively. Also, let ${A}_{i,j}^{a,b}$ denote the mean intensity value of the pixels located around ${p}_{i,j}$. In fact, ${A}_{i,j}^{a,b}$ can be computed through (1).

1 $${A}_{i,j}^{a,b}=\frac{\sum_{q=j-b}^{j+b}\sum_{p=i-a}^{i+a}{X}_{p,q}}{(2a+1)(2b+1)}$$

The pseudo-code for determining the boundary pixels of cysts is presented in Algorithm. 1. The main idea is that each pixel where a vertical intensity change and a diagonal intensity change occur can be considered as a boundary pixel for cyst. Therefore, the strategy is to verify whether or not vertical and diagonal intensity changes can be observed at each pixel. At first, the required parameters are initialized (line 1). Then, ${A}_{i,j}^{a,b}$ is computed for all ${p}_{i,j}$ s (line 2). Since the values of *a* and *b* are equal to 1 and 1, respectively, we should consider ${p}_{i,j}$ s with $2\le i\le m-1$ and $2\le j\le n-1$. In order to determine the points making significant vertical changes, we should compute ${dv}_{i,j}=({A}_{i-1,j}^{a,b}-{A}_{i+1,j}^{a,b})$ for all ${p}_{i,j}$ s (line 3). It should be mentioned that all pixels which provide local maximums for ${dv}_{i,j}$, make vertical intensity changes. Mathematically, the provision of local maximum for ${dv}_{i,j}$ is equivalent to simultaneously having ${dv}_{i,j}>{dv}_{i-1,j}$ and ${dv}_{i,j}>{dv}_{i+1,j}$. If the absolute value of such local maximums is more than a threshold denoted by *thv*, they correspond to significant vertical changes. Also, if the value of ${dv}_{i,j}$ is positive, a transition from light region to dark region occurs in ${p}_{i,j}$. In fact, ${p}_{i,j}$ can be considered as the entrance point for a possible cyst. Let *V* denote the set of all pixels with such characteristics (line 4) .
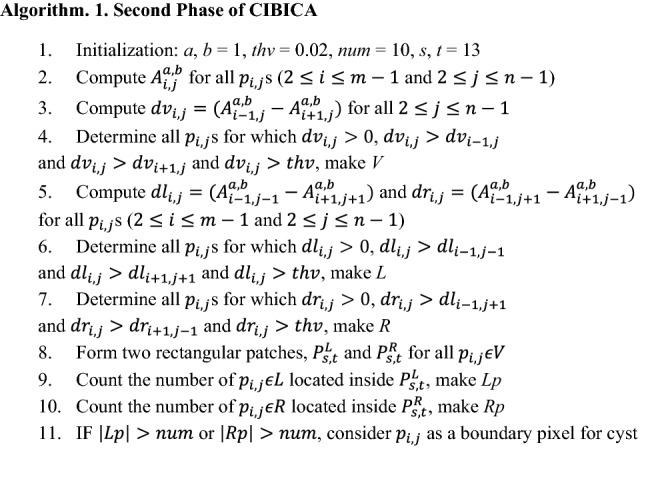


With respect to diagonal intensity change, it is necessary to compute ${dl}_{i,j}={A}_{i-1,j-1}^{a,b}-{A}_{i+1,j+1}^{a,b}$ for all ${p}_{i,j}$ s ($2\le i\le m-1$, $2\le j\le n-1$) to verify whether or not a diagonal intensity change occurs at the left side of ${p}_{i,j}$ (line 5). Each ${p}_{i,j}$ for which a local maximum can be observed in ${dl}_{i,j}$, makes a diagonal intensity change at the left side of ${p}_{i,j}$. The provision of local maximum in the left side of ${p}_{i,j}$ is equivalent to simultaneously have ${dl}_{i,j}>{dl}_{i-1,j-1}$, ${dl}_{i,j}>{dl}_{i+1,j+1}$. Also, we make sure that a significant intensity change has occurred at ${p}_{i,j}$ with ${dl}_{i,j}>thv$. Moreover, it is necessary to have ${dl}_{i,j}>0$ to make sure that a transition from light to dark region occurs (line 6). Let *L* denote the set of all points which satisfy the mentioned conditions.

In addition, we compute ${dr}_{i,j}={A}_{i-1,j+1}^{a,b}-{A}_{i+1,j-1}^{a,b}$ for all ${p}_{i,j}$ s ($2\le i\le m-1$, $2\le j\le n-1$) to determine whether or not a diagonal intensity change occurs at the right side of ${p}_{i,j}$ (line 5). Similar to the diagonal intensity change at the left side, it is necessary to simultaneously have ${dr}_{i,j}>0$, ${dr}_{i,j}>{dr}_{i-1,j+1}$, ${dr}_{i,j}>{dr}_{i+1,j-1}$ and ${dr}_{i,j}>thv$ for each ${p}_{i,j}$ to make sure a significant diagonal intensity change at the right side of it (line 7). The set of all pixels satisfying such conditions is denoted by *R*.

**Figure 3:**
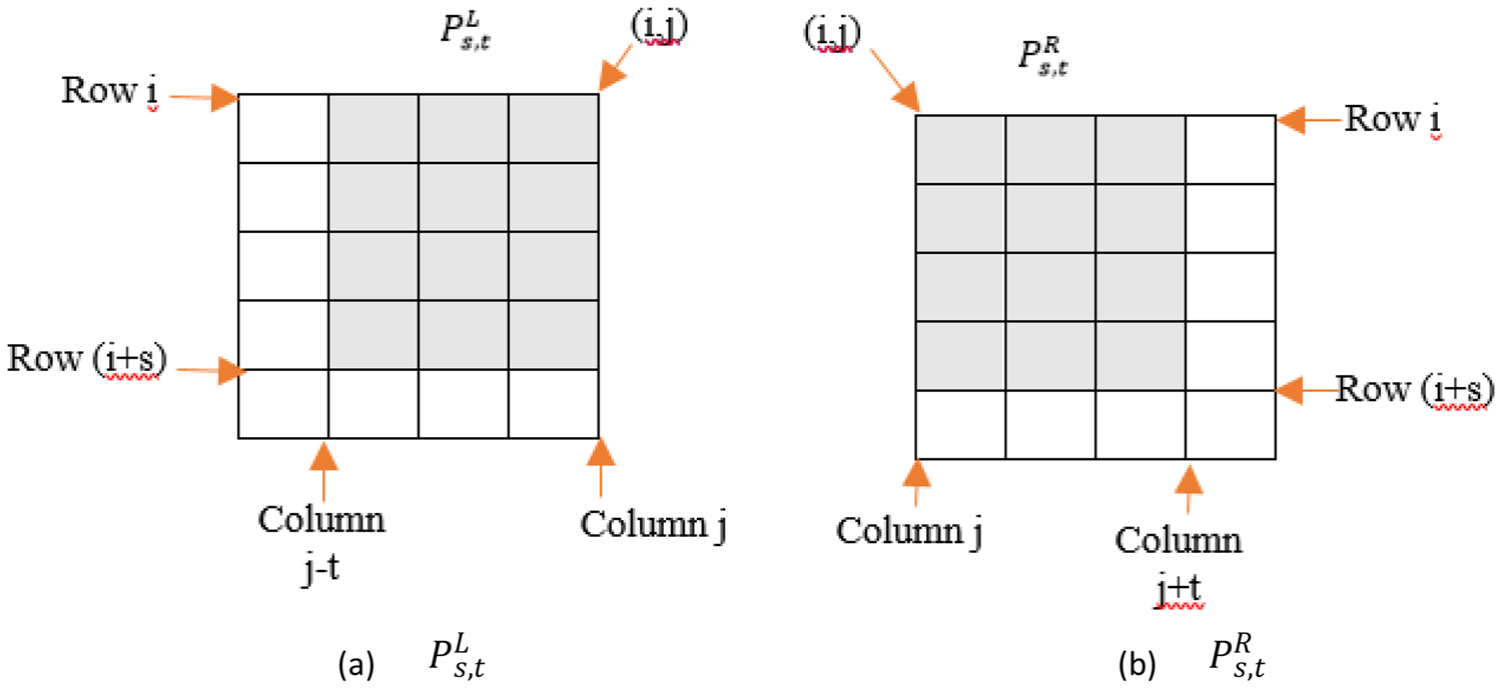
An example for presenting the locations of ${P}_{s,t}^{L}$ and ${P}_{s,t}^{R}$ around *p*_*i,j*_. The values of *s* and *t* are 4 and 3, respectively.

In order to make sure that both vertical and diagonal intensity change occur at one ${p}_{i,j}$, two rectangular patches are formed in the left and right sides of that pixel (line 8). Let ${P}_{s,t}^{L}$ and ${P}_{s,t}^{R}$ denote rectangular patches with *s* rows and *t* columns at the left and right sides of ${p}_{i,j}$, respectively. In Fig. 3, ${P}_{s,t}^{L}$ and ${P}_{s,t}^{R}$ are presented with gray squares around ${p}_{i,j}$. As can be observed in part a, ${P}_{s,t}^{L}$ is a rectangular patch located in the left side of *p*_*i,j*_ and the number of its rows and its columns are equal to *s* and *t*, respectively. Moreover, as obvious in part b, ${P}_{s,t}^{R}$ is a rectangular patch located in the right side of *p*_*i,j*_ and the number of its rows and its columns are equal to *s* and *t*, respectively. The number of pixels which belong to *L* and are located inside ${P}_{s,t}^{L}$ is counted (line 9). Let *Lp* denote the set of all pixels with such characteristics. If the number of members of *Lp* is more than a threshold value denoted by *num*, ${p}_{i,j}$ is considered as a boundary pixel for cyst (line 11). Similarly, the pixels belonging to *R* and are located inside ${P}_{s,t}^{R}$ are determined to make *Rp* which denotes the set of all pixels with such conditions (line 10). If the number of members of *Rp* is more than *num*, ${p}_{i,j}$ is considered as a boundary pixel for cyst (line 11).

### Post-processing

In this section, the superfluous pixels which are mistakenly extracted as the boundary pixels for cysts in the previous phase are removed. It should be mentioned that the existence of shadows in OCT images leads to have dark regions. The boundary pixels for such dark regions may be falsely considered as the boundary pixels for cysts. Therefore, if these points are not correctly identified and removed, the OCT image may be falsely interpreted as cystic. Thus, in this section a new algorithm is proposed for detecting shadow columns in the OCT image.

**Figure 4:**
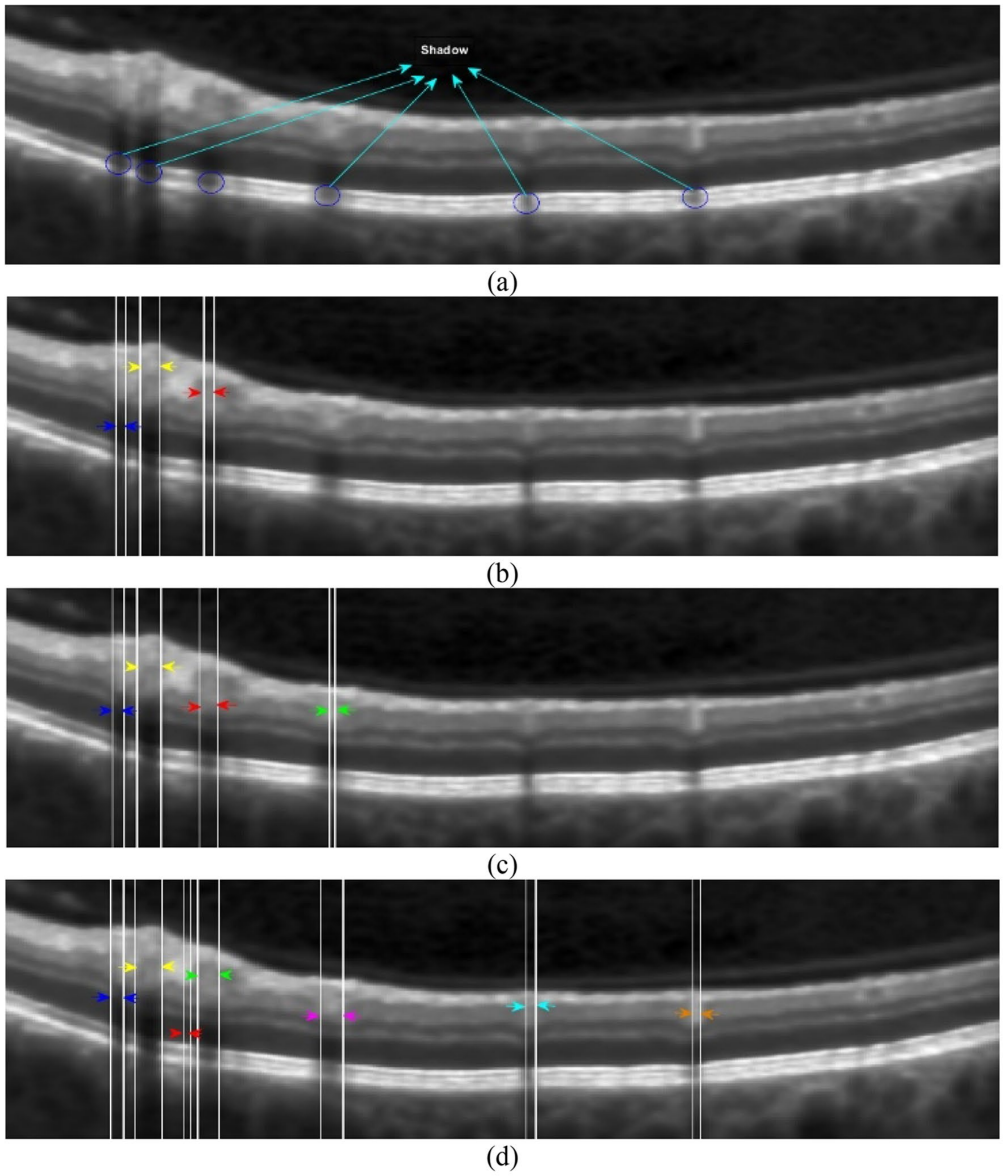
(**a**) An OCT image containing shadow columns, shadow columns extracted by the pseudo-code of Algorithm. 2 for (**b**) α = 0.16, (**c**) α = 0.12, (**d**) α = 0.08.

A sample OCT image which includes shadow columns is presented in Fig. 4a. Before explaining the new algorithm for shadow detection and the third phase of CIBICA algorithm, we notice to shadow regions in Fig. 4a to extract their distinguishing features. As can be observed in Fig. 4a, the shadow region is a set of consecutive dark columns. In fact, in the shadow regions, no significant vertical intensity change is observed after ONL + ISL layer boundary. This is while in the columns not including shadows, a significant intensity change is observed specially in the starting pixels of ONL + ISL layer or before RPE layer boundaries. This can be considered as a distinguishing feature between the columns including and not including shadow .
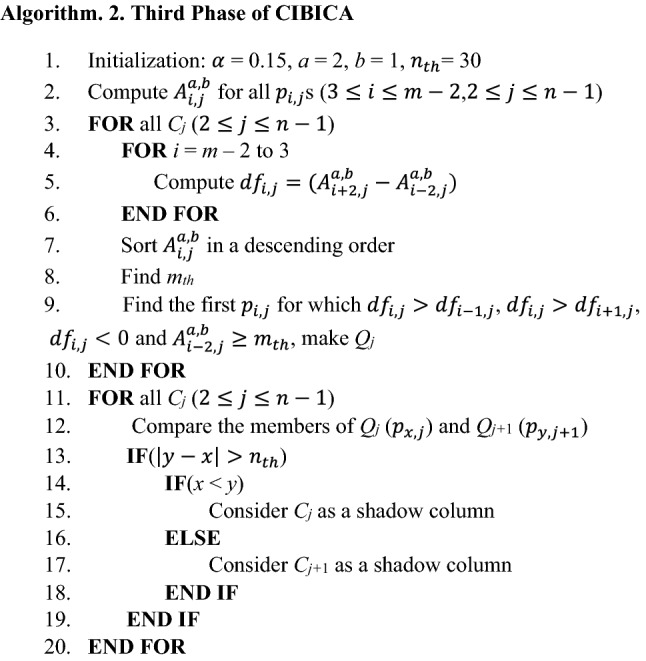

The pseudo-code for detecting shadow columns in the OCT image is presented in Algorithm. 2. Firstly, the required parameters are initialized (line 1). Then, the values of ${A}_{i,j}^{a,b}$ are computed for all ${p}_{i,j}$ s (line 2). Then, each column is separately verified to determine whether or not it is a shadow column (lines 3 to 10). For each ${p}_{i,j}$, the value of ${df}_{i,j}=({A}_{i+2,j}^{a,b}-{A}_{i-2,j}^{a,b})$ is computed (lines 4 to 6). It should be mentioned that in the pixels where the absolute values of ${df}_{i,j}$ are maximized, significant intensity changes occur. Then, since we look for a light region in the bottom of ONL + ISL boundary, we sort the values of ${A}_{i,j}^{a,b}$ in a descending order (line 7). We find *m*_*th*_ as the minimum value of ${A}_{i,j}^{a,b}$ among $[\alpha m]$ number of ${p}_{i,j}$ s with the highest values of ${A}_{i,j}^{a,b}$ (line 8). Then, we look for the most bottom ${p}_{i,j}$ that provides a local maximum for ${df}_{i,j}$ and a transition from dark to light regions (${df}_{i,j}<0$) (line 9). The light region should be light enough so that for which ${A}_{i-2,j}^{a,b}\ge {m}_{th}$ is true. Let ${Q}_{j}$ denote the first pixel for which such conditions are true. Then, a loop is written where the changes between the members of ${Q}_{j}$ ($2\le j\le n-1$) are evaluated (lines 12 to 20). It should be noticed that if there is a shadow in an arbitrary column, no significant intensity change from dark to light can be observed at the RPE layer boundary from bottom to top. The reason is that shadow is a dark region in which only light vertical transitions occur in the RPE layer boundary. Thus, if there is a significant change between the row indices of members of ${Q}_{j}$ in two consecutive columns, a shadow column exists there with high probability (lines 13 to 19).

It should be mentioned that the value of $\alpha$ affects the performance of the proposed method for detecting shadows. For the smaller values of $\alpha$, the light region where a significant transition from bottom to top occurs should be lighter than for bigger values of $\alpha$. The reason is that such a region should be among $[\alpha m]$ number of ${p}_{i,j}$ s with the highest values of ${A}_{i,j}^{a,b}$. Therefore, for the smaller values of $\alpha$, vertical intensity transitions after ONL + ISL boundary are identified under more difficult conditions. Thus, more columns are detected as shadow columns. This means that it may be possible to falsely identify several columns as shadow columns for the smaller values of $\alpha$. On the other hand, for the bigger values of $\alpha$, significant intensity changes from dark to light regions from bottom to top are easier detected in each column. Thus, less columns are identified as shadow columns. In fact, it is possible to miss several columns which are true shadow columns. Thus, it is important that what value is considered for $\alpha$. In Fig. 4b–d, it is possible to observe shadow columns between arrows with the same color for different values of $\alpha$. As can be observed in these parts, some true shadow columns are missed for the bigger value of $\alpha$ ($\alpha =$ 0.16, Fig. 4b). Also, some columns are mistakenly identified as shadow columns for the smaller value of $\alpha$ ($\alpha =$ 0.08, Fig. 4d). However, between the three values of 0.16, 0.12 and 0.08, $\alpha =$ 0.08 provides the best performance in the identification of shadow columns.

A method for detecting shadow areas in OCT Angiography (OCT-A) images is proposed in^36^. The method can identify shadows in both healthy and patient cases. The method of^36^ consists of pre-processing and shadow detection phases. In the pre-processing step, a directional graph search method is utilized to segment retinal layer interfaces. Then a thresholding method is used to remove noise pixels. Since OCT-A and OCT images are produced from the same optical signal, they are registered to provide helpful features for detecting shadows. In the shadow detection phase, four features which help in the process of shadow pixel identification are utilized. These features include local reflectance, local vessel density, reflectance standard deviation and local flow index and can be extracted from both OCT and OCT-A images. Then an ensemble classifier utilizes the extracted feature maps to segment shadowed areas. The machine learning method needs labeled training data.

## Experimental results

In this section, the numerical results obtained from the performance evaluation of CIBICA method are presented. The first dataset called KR-based dataset utilized for performance evaluation is the one previously used by^37^. This dataset is publicly online at http://people.duke.edu/~sf59/Chiu_BOE_2014_dataset.htm. This dataset consists of 110 OCT B-scans from 10 patients with severe DME pathology. We have also used another dataset called Kaggle dataset^38^. This dataset includes 108,312 images for four categories of OCT B-scans. These categories are DME, Drusen, Choroidal Neovascularization (CNV), and normal cases. From each DME and normal category, 242 images are selected for experiments.

In the pre-processing phase, we have also tried median filtering for de-noising in addition to SBSDI method which its execution time is much less than SBSDI. It should be mentioned that the accuracy metrics do not change when we use median filtering for de-noising purpose and the performance remains as same as before.

It should be mentioned that the computer system used for executing the proposed algorithm is a Core i7 with 6G RAM. The execution time for the second phase (boundary pixel determination) and the third phase (post-processing) is equal to 2 and 4.3 s for the images of Kaggle and KR-based datasets, respectively. The required time for de-noising is equal to 16 s in SBSDI method. It is observed that the results of the proposed method do not change when a different de-noising method is utilized. Using median filtering for de-noising, the total time of algorithm execution for three phases becomes equal to 4.2 and 6 s for the images of Kaggle and KR-based datasets.

It should be mentioned that using CIBICA method, we are able to determine a part of columns and rows that belong to cystic regions in OCT images. Also, we can extract some of boundary points for cysts. In other words, it may not be possible to extract all boundary points for cysts or all columns which belong to cystic regions, however, it is possible to predict whether or not the OCT B-scan includes any cystic region. This is the reason why CIBICA method is applicable for the purpose of detecting cystic images.

**Figure 5:**
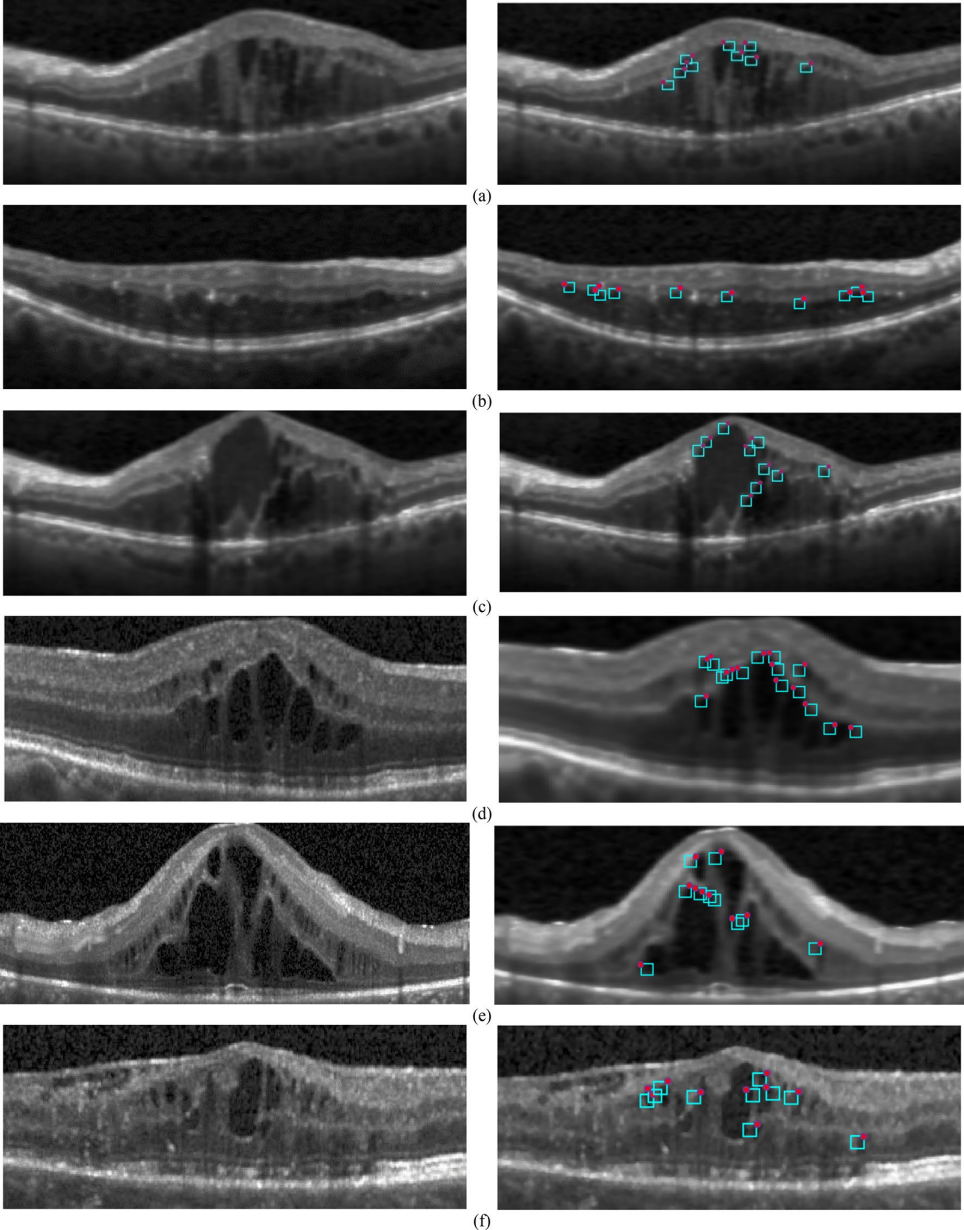
(**a**–**c**) left parts: Sample OCT B-scans from KR-based dataset, right parts: extracted cystic regions, (**d**–**f**) left parts: Sample OCT B-scans from Kaggle dataset, right parts: extracted cystic regions.

The performance of the proposed algorithm for extracting cystic regions is presented in Fig. 5. In this figure several OCT B-scans from the two datasets are shown and the region cystic extracted by the proposed method are also presented. Parts (a), (b), and (c) are from KR-based dataset and Parts (e), (f) and (g) are from Kaggle dataset. It should be mentioned that for each image which is located in the left side, the extracted cystic regions by the CIBICA method are presented in the right figure. The extracted cystic regions have been shown with blue squares. Also, for each blue square, we have presented one pixel with red circle which shows the first candidate pixel for that square. Here, we consider that pixel as *p*_*i,j*_. In drawing blue boxes, we notice that which of ${P}_{s,t}^{L}$ and ${P}_{s,t}^{R}$ around the extracted *p*_*i,j*_ has the adequate number of pixels with considerable intensity changes in the diagonal direction. If ${P}_{s,t}^{L}$ has the sufficient pixels with such conditions, then a blue box is drawn in the left side of *p*_*i,j*_. The dimensions of this box are equal to *s* and *t*. In fact, the number of rows for this box is equal to *s* and the number of columns is equal to *t*. On the other hand, if ${P}_{s,t}^{R}$ has the sufficient pixels with significant intensity change in the diagonal direction, then a blue box is drawn in the right side of *p*_*i,j*_. Figure 3 indicates how ${P}_{s,t}^{L}$ and ${P}_{s,t}^{R}$ are located around *p*_*i,j*_. It should be noted that all the pixels with intensity changes in the diagonal directions are the boundary pixels for cyst. Since we have considered the value of 10 for num, for each blue square at least we have 11 boundary pixels for cyst including the first pixel which is shown with red circle. Also, the average number of boundary pixels for each blue square is equal to 12. It should be mentioned that it is possible to increase the number of the cystic regions extracted by the proposed method. This can be possible through regulating the value of parameters which will be discussed later in this section.

Table 1 presents the values of performance metrics utilized for evaluating the accuracy of the proposed method. The first metric is sensitivity (*SE*) which determines the ratio of true positive cases to the sum of all true positive (*TP*) and false negative (*FN*) cases. The second metric is specificity (*SP*) determining the ratio of true negative (*TN*) cases to the sum of all true negative and false positive (*FP*) cases. In fact, (2) and (3) are true for sensitivity and specificity, respectively.

**Table 1:** The summary of *SE* and *SP* parameters for CIBICA method for KR-based and Kaggle datasets.

Parameter		SE (KR-based)	SP(KR-based)	SE (Kaggle)	SP (Kaggle)
α = 0.1	*s*, *t* = 13	0.79	0.74	0.88	0.8
α = 0.1	*s*, *t* = 11	0.91	0.61	0.92	0.73
α = 0.15	*s*, *t* = 13	0.86	0.57	0.9	0.7
α = 0.15	*s*, *t* = 11	0.92	0.39	0.93	0.62

2 $$SE=\frac{TP}{TP+FN}$$

3 $$SP=\frac{TN}{TN+FP}$$

It should be noticed that having high values for both *SE* and *SP* is considerably important. The reason is that both parameters measures the capability of method in correctly interpreting an image as cystic or non-cystic. For instance, if the value of *SE* is high and the value of *SP* is low, it means that although the method is good at truly localization the cysts (*TP* is high), but it is not good at correctly detecting no-cystic areas (*TN* is low).

The values of *SE* and *SP* for CIBICA method for the two datasets are presented in Table 1. It should be noted that for different values of α and *s* and *t*, different values are obtained for *SE* and *SP*. In fact, such parameters affect the performance of the proposed method. As can be observed in the table, the value of *SE* for KR-based dataset ranges from 0.79 to 0.92 for different combinations of α and *s*, *t*. It should be mentioned that if we consider a constant value for window size (*s* and *t*), the value of *SE* increases with increment in the value of *α*. For instance, the value of *SE* reaches to 0.86 from 0.79 with increment of α from 0.1 to 0.15 (*s*, *t* = 13). The reason is that when the value of α increases, less number of columns are detected as shadow columns and more points are identified as boundary points for cysts. This leads to increment in the number of TPs and consequently increased *SE*. Also, increasing the value of α from 0.1 to 0.15 leads to decrement in the value of *SP* from 0.74 to 0.57. The reason is that the less number of columns are identified as shadow columns under such a change and some true shadow columns are considered as non-shadow columns. Therefore, the boundary points which are located on such columns are mistakenly considered as boundary points for cysts. Thus, the number of FPs increases and consequently the value of *SP* is reduced under such conditions.

In addition, if we consider a constant value for α, the decrement in the window size leads to the increment in the value of *SE*. For instance, if we reduce the window size from *s*, *t* = 13 to *s*, *t* = 11 (α = 0.15), the value of *SE* increases from 0.86 to 0.92. The reason is that in the windows with smaller sizes, it is easier to find a number of pixels with significant diagonal intensity changes. Thus, it is possible to identify true boundary points for cysts with high probability. However, under such conditions, the pixels which are boundary points for layers and not boundary points for cysts may be mistakenly considered as the boundary pixels for cysts. Therefore, the number of FPs also increases and the value of *SP* is reduced.

With respect to the results of Kaggle dataset, the value of *SE* ranges from 0.88 to 0.93 for different combinations of α and *s*, *t*. If we consider a constant value for window size (*s* and *t*), the value of *SE* increases with increment in the value of *α*. For instance, the value of *SE* reaches to 0.9 from 0.88 with increment of α from 0.1 to 0.15 (*s*, *t* = 13). In addition, if we consider a constant value for α, the decrement in the window size leads to the increment in the value of *SE*. For instance, if we reduce the window size from *s*, *t* = 13 to *s*, *t* = 11 (α = 0.15), the value of *SE* increases from 0.9 to 0.93.

For KR-based dataset, we have compared the results of performance evaluation of CIBICA method with the Kernel-Regression (KR) based method of^37^. In the KR-based method of^37^, seven retinal layers and fluid-filled regions are identified using a kernel regression classification method. KR is a nonparametric mathematical model which can be used to derive the local estimates of a function with the help of a kernel. Such a kernel can weigh the relative importance of nearby points. In the KR-based method of^37^, the locations of retinal layers and fluid regions are firstly estimated. Then, these estimates are utilized for the segmentation of retinal layers and fluid-filled regions using a graph theory and dynamic programming framework.

The values of *SP* and *SE* for KR-based method of^37^ are equal to 0.52 and 0.79, respectively. In order to compare the performance of our proposed method with the KR-based method of^37^, it should be noted that the least value of *SE* in our proposed method is 0.79 which corresponds to the value of 0.74 for *SP*. Since 0.74 is much more than 0.52, the better performance of our proposed method is confirmed. The other values obtained for *SE* including 0.91 and 0.86 correspond to 0.61 and 0.57 for *SP* values which are better than that of KR-based method.

Supplementary 1 presents several OCT B-scans from the dataset of^37^ to visually compare the capability of the proposed CIBICA method and KR-based method of^37^ in cyst detection. The OCT B-scans in part a are totally labeled as the B-scans including cystic regions by the ophthalmologist. The KR-based method of^37^ falsely identified no cystic region in the B-scans of this part while the proposed CIBICA method has correctly identified all of them as the B-scans consisting of cysts. The cystic regions extracted by CIBICA method for the images of part a are presented in part c. The extracted cystic regions are presented with blue squares. Also, the first candidate pixels extracted in the second phase are shown with red circles. This can clarify that the KR-based method of^37^ cannot detect cystic regions with small heights. The reason is that all the sample B-scans in part a include cysts with small heights which are separated from surface 6 of retinal layers (presented in Fig. 1). The cystic regions labeled by the ophthalmologists are presented with white regions in part b. Thus, it can be concluded that the proposed CIBICA method has better performance in detecting small cysts which are slightly separated from the borders of retinal layers.

In addition, the B-scans in part d are totally labeled as the B-scans not including cystic regions by the ophthalmologist. The KR-based method of^37^ falsely identified them as the B-scans containing cysts. One reason is that the method of^31^ cannot identify shadow columns in the OCT B-scans and the regions near to shadow columns are mistakenly interpreted as cysts. However, the proposed CIBICA method is capable of correctly identifying all the B-scans of part d as the B-scans not including cysts. The main reason is that the proposed CIBICA method contains an important part for detecting shadow columns and removing the boundary pixels located near them. This leads to the decrement in the false positive cases in the CIBICA method and increment in the specificity.

## Conclusions

In this paper, a new method is proposed to detect cystoid OCT B-scans. The method includes three phases which are pre-processing, boundary pixel determination and post-processing. In the pre-processing phase, a de-noising method is applied on the image to prepare it for processing. In the boundary pixel determination phase, boundary pixels for cysts are detected and a novel feature for them is extracted. In this phase, the new method separately verifies each column to determine whether or not it includes any pixel at which a significant intensity change occurs. Also, for such pixels, a rectangular patch is considered at both left and right sides and the number of pixels which make significant diagonal intensity changes in each patch is measured. If the number of such pixels is sufficient, the first pixel can be a candidate boundary pixel for a cystoid region. In other words, in the boundary pixel determination phase, the directional analysis of intensity changes is performed. The reason is that cysts can be observed as intensity changes in several directions. It should be mentioned that some of the extracted boundary pixels may be located on the boundaries of shadow regions and therefore they were mistakenly extracted. Thus, in the post-processing phase, a novel algorithm is proposed to correctly detect the shadow columns in the OCT B-scan. This helps in the omission of superfluous points which are located in the shadow columns and are mistakenly extracted as the boundary pixels for cysts. The performance of the proposed method has been evaluated on a real dataset containing 110 B-scans from 10 patients. The numerical results show that the proposed method produces improved accuracy in a significantly short period of time. In future, the proposed method can be developed to be useful for the exact segmentation and quantification of cysts. Also, it can be extended for the separation and classification of different types of cysts including SRF and IRF.

## Supplementary Information

Supplementary Figure 1.

## References

[1] Fujimoto JG, Drexler W, Schuman JS, Hitzenberger CK (2009). Optical coherence tomography (OCT) in ophthalmology: Introduction. Opt. Express..

[2] Fujimoto JG, Pitris C, Boppart SA, Brezinski ME (2000). Optical Coherence Tomography: An emerging technology for biomedical imaging and optical biopsy. Neoplasia.

[3] Monemian M, Rabbani H (2020). Analysis of a novel segmentation algorithm for optical coherence tomography images based on pixels’ intensity correlations. IEEE Trans. Instrum. Meas..

[4] Monemian M, Rabbani H (2020). Mathematical analysis of texture indicators for the segmentation of optical coherence tomography images. Optik.

[5] Abramoff MD, Garvin M, Sonka M (2010). Retinal imaging and image analysis. IEEE Rev. Biomed. Eng..

[6] Wilkins GR, Houghton OM, Oldenburg AL (2012). Automated segmentation of intra-retinal cystoid fluid in optical coherence tomography. IEEE Trans. Biomed. Eng..

[7] Esmaeili M, Dehnavi AM, Rabbani H, Hajizadeh F (2016). 3D segmentation of retinal cysts from SD-OCT images by the use of three-dimensional curve-let based K-SVD. J. Med. Signals Sens..

[8] Girish GN, Thakur B, Roychowdhury S, Kothari AR, Rajan J (2017). Segmentation of intra-retinal cysts from optical coherence tomography images using a fully convolutional neural network model. IEEE J. Biomed. Health. Inform..

[9] Gopinath K, Sivaswamy J (2018). Segmentation of retinal cysts from Optical Coherence Tomography volumes via selective enhancement. IEEE J. Biomed. Health. Inform..

[10] Quellec G, Lee K, Dolejsi M, Garvin MK, Abramoff MD, Sonka M (2010). Three-dimensional analysis of retinal layer texture: Identification of fluid-filled regions in SD-OCT of the macula. IEEE Trans. Med. Imaging..

[11] de Moura J, Vidal PL, Novo J, Rouco J, Penedo MG, Ortega M (2020). Intra-retinal fluid pattern characterization in optical coherence tomography images. Sensors..

[12] Chen X, Niemeijer M, Zhang L, Lee K, Abramoff MD, Sonka M (2012). Three-dimensional segmentation of fluid-associated abnormalities in retinal oct: Probability constrained graph-search-graph-cut. IEEE Trans. Med. Imaging..

[13] Fernandez DC (2005). Delineating fluid-filled region boundaries in optical coherence tomography images of the retina. IEEE Trans. Med. Imaging..

[14] Wang J, Zhang M, Pechauer AD, Liu L, Hwang TS, Wilson DJ, Li D, Jia Y (2016). Automated volumetric segmentation of retinal fluid on optical coherence tomography. Biomed. Opt. Express..

[15] Montuoro A, Waldstein SM, Gerendas BS, Erfurth US, Bogunovic H (2017). Joint retinal layer and fluid segmentation in OCT scans of eyes with severe macular edema using unsupervised representation and auto-context. Biomed. Opt. Express..

[16] Roy AG, Conjeti S, Karri SPK, Sheet D, Katouzian A, Wachinger C, Navab N (2017). ReLayNet: Retinal layer and fluid segmentation of macular optical coherence tomography using fully convolutional networks. Biomed. Opt. Express..

[17] Venhuizen FG, Ginneken BV, Liefers B, Asten FV, Schreur V, Fauser S, Hoyng C, Theelen T, Sanchez CI (2018). Deep learning approach for the detection and quantification of intra-retinal cystoid fluid in multivendor optical coherence tomography. Biomed. Opt. Express..

[18] Terry L, Trikha S, Bhatia KK, Graham MS, Wood A (2021). Evaluation of automated multiclass fluid segmentation in optical coherence tomography images using the Pegasus fluid segmentation algorithms. Transl. Vis. Sci. Technol..

[19] Vidal PL, Moura JD, Novo J, Penedo MG, Ortega M (2018). Intra-retinal fluid identification via enhanced maps using optical coherence tomography images. Biomed. Opt. Express..

[20] Bogunovic H, Venhuizen F, Klimscha S, Apostolopoulos S, Hadiashar AB (2019). RETOUCH: The retinal OCT fluid detection and segmentation benchmark and challenge. IEEE Trans. Med. Imaging..

[21] Zheng Y, Sahni J, Campa C, Stangos AN, Raj A, Harding SP (2013). Computerized assessment of intra-retinal and sub-retinal fluid regions in spectral-domain optical coherence tomography images of the retina. Am. J. Ophthalmol..

[22] Xu X, Lee K, Zhang L, Sonka M, Abràmoff MD (2015). Stratified sampling Voxel classification for segmentation of intra-retinal and sub-retinal fluid in longitudinal clinical OCT data. IEEE Trans. Med. Imaging..

[23] Rashno A, Nazari B, Koozekanani DD, Drayna PM, Sadri S, Rabbani H, Parhi KK (2017). Fully-automated segmentation of fluid regions in exudative age-related macular degeneration subjects: Kernel graph cut in neutrosophic domain. PLoS ONE.

[24] Rashno A, Koozekanani DD, Drayna PM, Nazari B, Sadri S, Rabbani H, Parhi KK (2018). Fully automated segmentation of fluid/cyst regions in optical coherence tomography images with diabetic macular edema using neutrosophic sets and graph algorithms. IEEE Trans. Biomed. Eng..

[25] Schlegl T, Waldstein SM, Bogunovic H, Endstraber F, Sadeghipour A, Philip AM, Podkowinski D, Gerendas BS, Langs G, Erfurth US (2018). Fully automated detection and quantification of macular fluid in OCT using deep learning. Ophthalmology.

[26] Kang, S. H., Park, H. S., Jang, J. & Jeon, K. Deep neural networks for the detection and segmentation of the retinal fluid in OCT images. In *RETOUCH.* 9–14 (2017).

[27] Oguz, I., Zhang, L., Abràmoff, M. D. & Sonka, M. Optimal retinal cyst segmentation from OCT images. In *Proc. SPIE 9784, Medical Imaging* (2016).

[28] The Iowa Reference Algorithms. (Iowa Inst. Biomed. Imag.) http://www.biomed-imaging.uiowa.edu/downloads/.

[29] Sacconi R, Lutty GA, Mullins RF, Borrelli E, Bandello F, Querques G (2019). Subretinal pseudocysts: A novel OCT finding in diabetic macular edema. Am. J. Ophthalmol. Case Rep..

[30] Guo Y, Hormel TT, Xiong H, Wang J, Hwang TS, Jia Y (2020). Automated segmentation of retinal fluid volumes from structural and angiographic optical coherence tomography using deep learning. Transl. Vis. Sci. Technol..

[31] Wolff B, El Maftouhi MQ, Faysse MM (2010). Retinal cysts in age-related macular degeneration: An OCT study. Investig. Ophtalmol. Vis. Sci..

[32] Montuoro A, Waldstein SM, Gerendas BS, Schmidt-Erfurth U, Bogunović H (2017). Joint retinal layer and fluid segmentation in OCT scans of eyes with severe macular edema using unsupervised representation and auto-context. Biomed. Opt. Express..

[33] Venhuizen FG, van Ginneken B, Liefers B, van Asten F, Schreur V, Fauser S, Hoyng C, Theelen T, Sánchez CI (2018). Deep learning approach for the detection and quantification of intraretinal cystoid fluid in multivendor optical coherence tomography. Biomed. Opt. Express.

[34] Lv H, Fu S, Zhang C, Zhai L (2018). Speckle noise reduction of multi-frame optical coherence tomography data using multi-linear principal component analysis. Opt. Express..

[35] Fang L, Li S, McNabb RP, Nie Q, Kuo AN, Toth CA, Izatt JA, Farsiu S (2013). Fast acquisition and reconstruction of optical coherence tomography images via sparse representation. IEEE Trans. Med. Imaging..

[36] Camino A, Jia Y, Yu J, Wang J, Liu L, Huang D (2019). Automated detection of shadow artifacts in optical coherence tomography angiography. Biomed. Opt. Express.

[37] Chiu SJ, Allingham MJ, Mettu PS, Cousins SW, Izatt JA, Farsiu S (2015). Kernel regression-based segmentation of optical coherence tomography images with diabetic macular edema. Biomed. Opt. Express..

[38] Kermany DS, Goldbaum M, Cai W, Anthony Lewis M, Xia H, Zhang K (2018). Identifying medical diagnosis and treatable diseases by image-based deep learning. Cell Resour..

