# Supplementary material for: Directional analysis of intensity changes for determining the existence of cyst in optical coherence tomography images
Source: Sci Rep. 2022 Feb 8;12:2105. doi: 10.1038/s41598-022-06099-6 (PMC8825816; doi:10.1038/s41598-022-06099-6)
Supplement: Supplementary file 1 — Supplementary Figure 1. [file 41598_2022_6099_MOESM1_ESM.docx]

Supplementary Information

Title of article: Red-lesion Extraction in Retinal Fundus Images by Directional Intensity

Changes’ Analysis

Authors: Maryam Monemian and Hossein Rabbani

Corresponding author: Hossein Rabbani, Medical Image & Signal Processing Research

Center, School of Advanced Technologies in Medicine, Isfahan University of Medical Sciences,

Isfahan, Iran, 8174673461, email: Rabbani.h@gmail.com

**Supplementary Information**

**Title of article:** Directional Analysis of Intensity Changes for Determining the Existence of Cyst in Optical Coherence Tomography Images

**Authors:** Maryam Monemian and Hossein Rabbani

**Corresponding author:** Hossein Rabbani, Medical Image & Signal Processing Research Center, School of Advanced Technologies in Medicine, Isfahan University of Medical Sciences, Isfahan, Iran, 8174673461, email: [Rabbani.h@gmail.com](mailto:Rabbani.h@gmail.com)

| 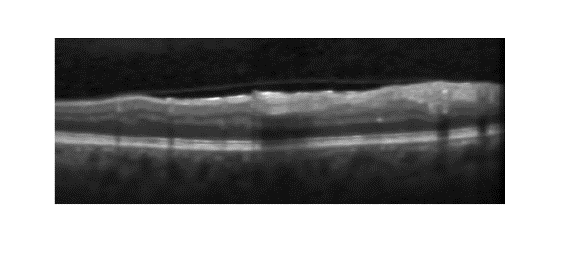 | 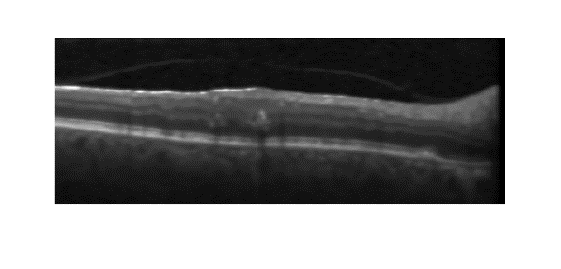 | 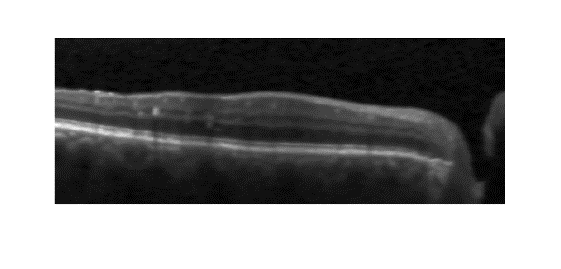 |
| --- | --- | --- |
| (a) | | |
| 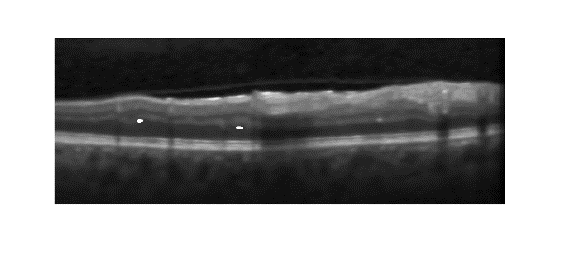 | 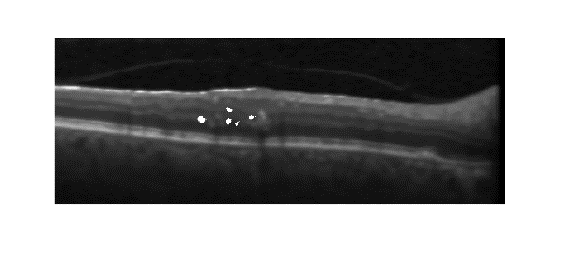 | 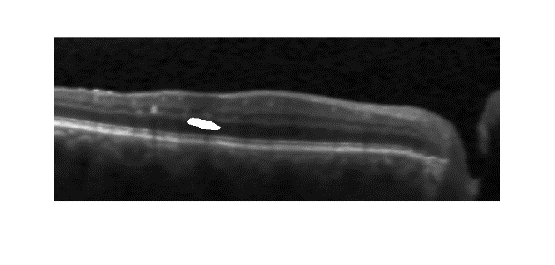 |
| (b) | | |
| 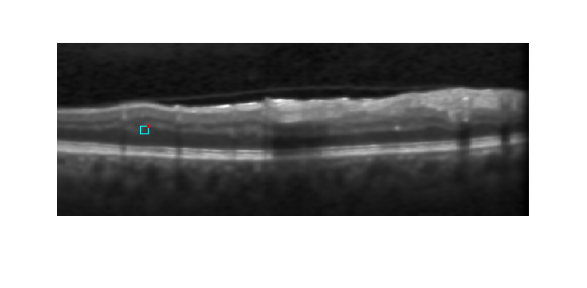 | 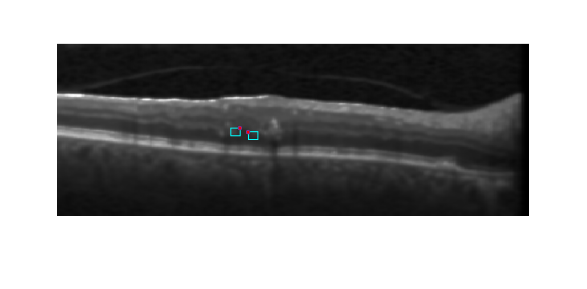 | 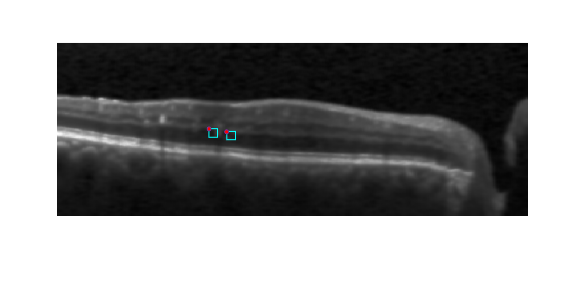 |
| (c) | | |
| 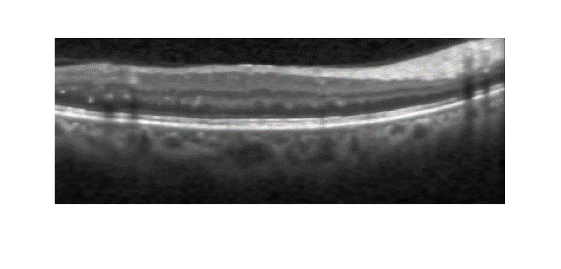 | 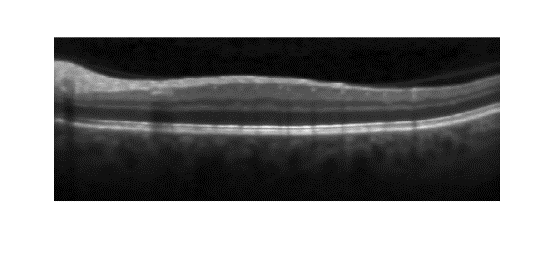 | 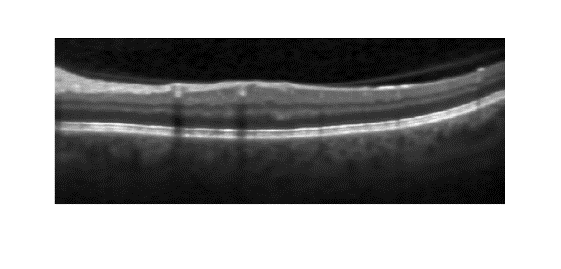 |
| (d) | | |
| 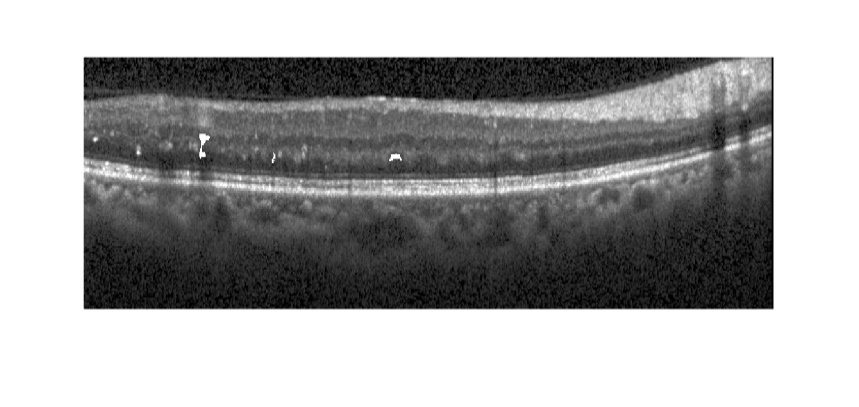 | 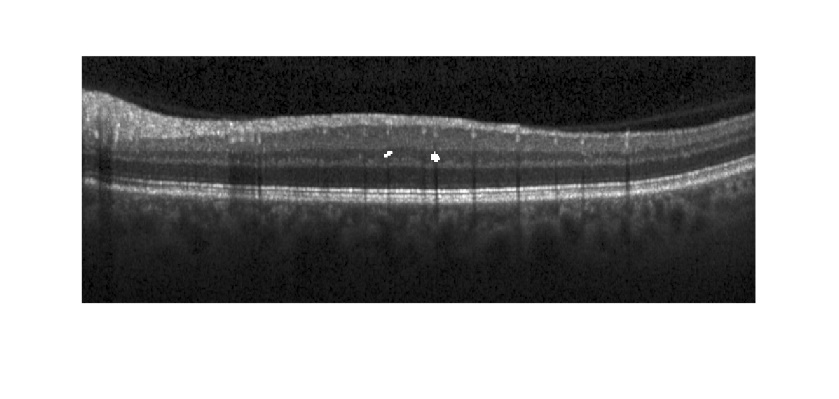 | 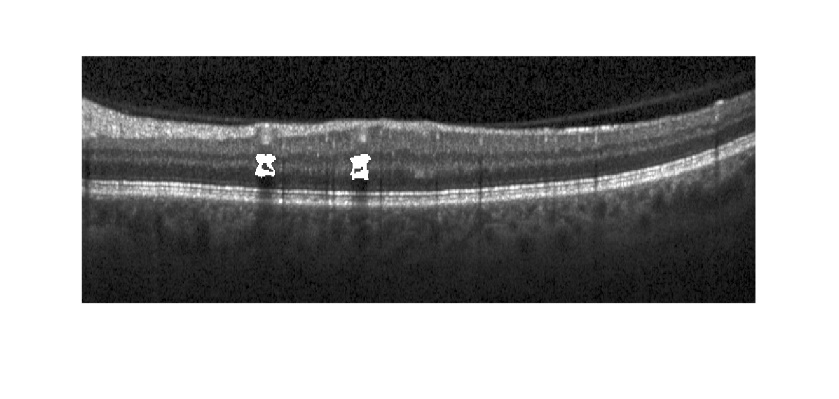 |
| (e) | | |

Supplementary. 1. Representative OCT B-scans, a) labeled as including cysts by the ophthalmologist, b) cystic regions of figures of part a, c) cystic regions extracted by CIBICA method, d) B-scans labeled as without cyst by the ophthalmologist and e) the results of KR-based method on B-scans (d) which segments cystic regions (white areas) by mistake.
